# Task Complexity Differentially Affects Executed and Imagined Movement Preparation: Evidence from Movement-Related Potentials

**DOI:** 10.1371/journal.pone.0009284

**Published:** 2010-02-19

**Authors:** Cornelia Kranczioch, Simon Mathews, Philip Dean, Annette Sterr

**Affiliations:** 1 Department of Neurology, University Hospital Jena, Jena, Germany; 2 Department of Psychology, University of Surrey, Guildford, United Kingdom; 3 Department of Psychology, Carl von Ossietzky University Oldenburg, Oldenburg, Germany; 4 Department of Psychology, University of Portsmouth, Portsmouth, United Kingdom; University of Regensburg, Germany

## Abstract

**Background:**

The neural simulation theory predicts similarity for the neural mechanisms subserving overt (motor execution) and covert (movement imagination) actions. Here we tested this prediction for movement preparation, a key characteristic of motor cognition.

**Methodology/Principal Findings:**

High-density electroencephalogram (EEG) was recorded during covert and overt actions. Movement preparation was studied with a motor priming paradigm, which varied task complexity and amount of advance information. Participants performed simple or complex sequential finger movements either overtly or covertly. Advance information was either fully predictive or partially predictive. Stimulus-locked event-related potential (ERP) data showed the typical pattern of foreperiod activation for overt and covert movements. The foreperiod contingent negative variation (CNV) differed between simple and complex movements only in the execution task. ERP topographies differed between execution and imagination only when advance information was fully predictive.

**Conclusions/Significance:**

Results suggest a differential contribution of the movement preparation network to action imagination and execution. Overt and covert actions seem to involve similar though not identical mechanisms, where overt actions engage a more fine-grained modulation of covert preparatory states.

## Introduction

According to the neural simulation theory [1] actions are not confined to an overt stage but also contain a covert stage. The model claims that ‘covert actions are in fact actions that are not executed’ and that ‘covert actions are neurally simulated actions’ (p.103). In the case of motor imagery, this assumption predicts similarity for the neural mechanisms subserving action imagination and action execution. Supportive evidence for this claim, primarily obtained through functional Magnetic Resonance Imaging (fMRI) studies, suggests that motor imagery and overt motor execution share many commonalities in terms of performance and underlying neural substrates (for reviews see [1], [2]). An open question however concerns the processes involved in the preparation for actions, a key characteristic of motor cognition. In a recent fMRI study Hanakawa and colleagues [3] found that preparatory brain activity is more similar though not identical to movement imagery than to movement execution. However, similar to earlier fMRI research [4] a direct comparison of preparatory processes for imagination and execution was not possible with the chosen design. In addition, the preparatory phase was rather long (12–18 seconds) because of the slow time course of the blood oxygenation level dependent (BOLD) response. Participants therefore had time to engage in a range of covert activities, which very likely also included imagery of the upcoming task.

Because of the timing problem inherent to all measures relying on haemodynamic measures, preparatory brain activity is best investigated with methods of high temporal resolution, such as the electroencephalogram (EEG). A classic approach to study preparatory motor activity that is also suitable for the EEG environment is the motor priming paradigm. Here, a prime stimulus (S1) conveys information about particular aspects of an upcoming movement cued by the response cue (S2) [5]. In the S1–S2 interval (the foreperiod), an event-related potential (ERP), the contingent negative variation (CNV), is observed over central scalp locations. Critical for motor preparation is the late CNV in the last ∼500 ms prior to S2 presentation which is an index of preparation and motor processing, but which is also associated with judgment, estimation and cognition [6], [7].

Preparatory activity has been extensively investigated in motor execution paradigms using ERPs e.g. [6], [7], [8], [9], [10] but only few experiments have applied the ERP method to study motor preparation in imagery. Initial evidence suggests that preparatory ERP waveforms for imagined and executed movements are reasonably similar in the early preparatory phase but show reduced amplitudes for imagined movements in the later stages of the preparatory phase [11]–[13]. The latter presumably results from reduced activation of the primary motor cortex (M1) in imagery. Studies on the lateralised readiness potential (LRP) [14], [15], an index of motor-related, lateralised aspects of preparatory activity, provide further evidence for functional equivalence between imagination and execution in the preparatory phase, but, similar to the CNV, show attenuated LRP amplitude for imagined movements. This leads Carillo-de-la-Pena et al. [15] to conclude that motor execution and motor imagery are “equivalent” but not “equal”.

The functional similarity of advance movement preparation in motor execution and imagery is of great importance for applied fields such as rehabilitation and sports, and more research on this question is therefore needed. The existing evidence is suggestive, however, none of the studies have directly compared preparatory indices for overt (execution) and covert (imagined) movements within one experiment. Furthermore, a recent study by Schröter and Leuthold [16] (see also [6]) suggests that not only the amount of advance information [8]–[10] but also the complexity of the anticipated movement modulates preparatory ERP makers in an execution paradigm. Whether this association is also true for imagined movements is unclear. The present study therefore aimed to (1) affirm the notion of functional similarity for preparatory activity during the anticipation of overt (executed) and covert (imagined) movements when the two different modes are compared within the same experiment, and (2) test the hypothesis that the assumption of similarity during motor preparation extends to task complexity.

In the present study we therefore compared the preparation for covert and overt actions in an S1–S2 paradigm using high-density EEG while manipulating both task complexity and amount of advance information. Based on previous research we expected an attenuation of the late CNV during preparation for motor imagery as compared to motor execution. Furthermore, and in accordance with the neural simulation theory, we anticipated a high degree of functional similarity between the two conditions that would be reflected in similar effects of advance information and task complexity on CNV amplitude. Specifically, an increase in movement complexity, as well as a higher amount of advance information, was expected to result in an increase in CNV amplitude irrespective of preparation modality (overt or covert action).

## Methods

### Subjects

Twelve right-handed volunteers (four male, mean age 24.6, SD 5.6) participated in two two-hour recording sessions. Sessions were conducted on consecutive days. An hourly rate of £5 was paid for participation, plus a £5 bonus for good adherence to task instructions. The study was approved by the University of Surrey research ethics committee and complied with the Declaration of Helsinki. Written informed consent was taken prior to participation. All participants had normal or corrected-to-normal vision.

### Procedure

**Figure 1:**
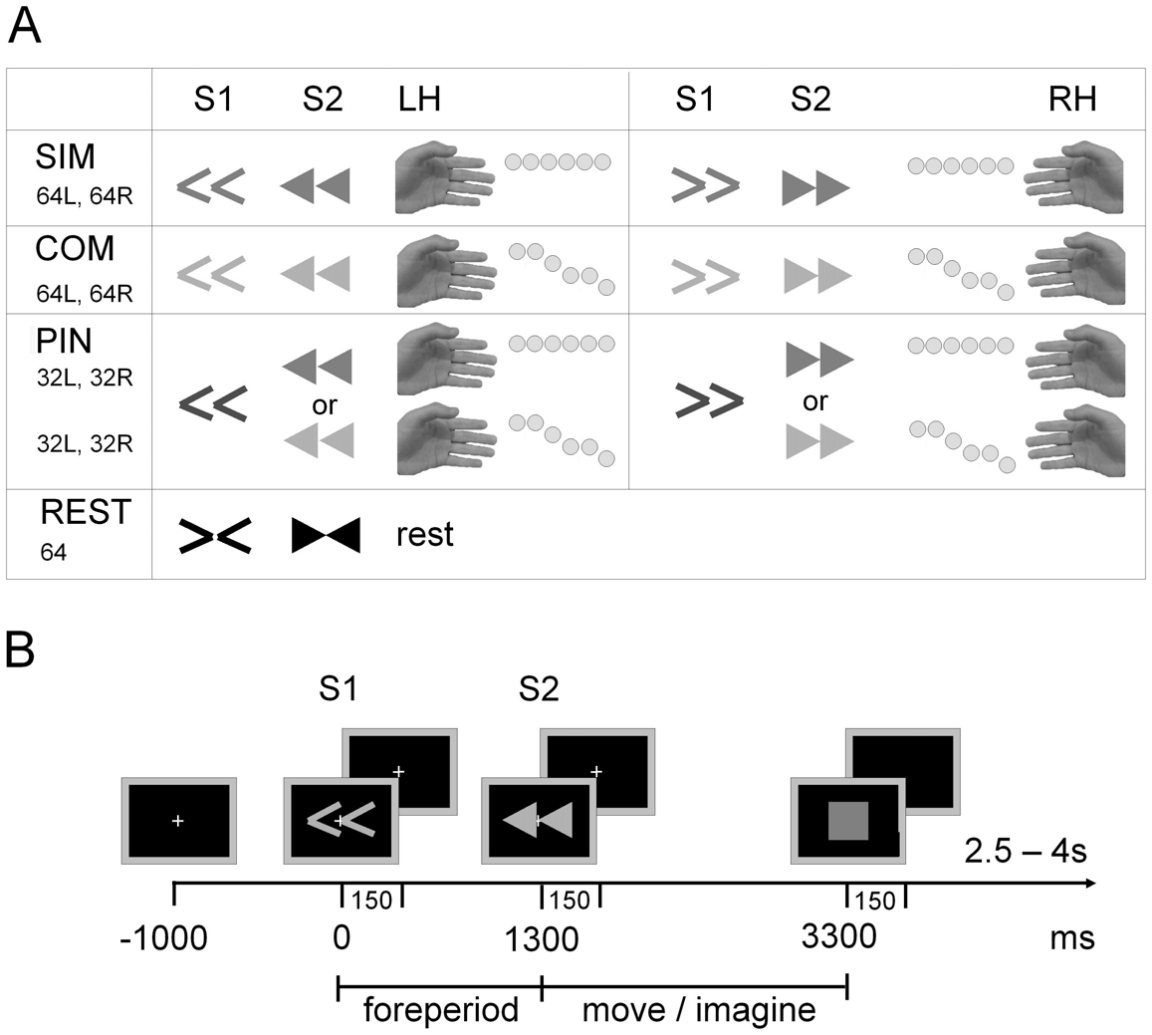
Experimental design. (A) Experimental conditions and associated stimuli. S1 and S2 arrow stimuli are shown followed by the required tapping sequence. Shades of grey represent different arrow colours (blue, pink and green) per condition (counterbalanced across participants). Total trial counts are shown below the condition name for left (L) and right (R) hands. Grey dots next to the fingers indicate the thumb-finger tap sequence in each condition. (B) An example trial sequence showing a complex left hand trial.

Participants sat in a dimly lit room at a viewing distance of 70 cm from a screen. They placed their hands in a relaxed, comfortable position on the desk in front of them with their palms faced upwards. Hand position was chosen as to avoid tactile stimulation of the fingertips by the desk. Trials began with a 1 s presentation of a central fixation cross after which S1 was presented to instruct participants to prepare for the overt execution (execution session) or the imagination (imagination session) of sequential finger-thumb oppositions. This preparatory period was 1300 ms, after which the imperative stimulus (S2) was presented to cue the movement or imagination onset. A varying time interval (2.5 s to 4 s) elapsed before presentation of the next trial. Trial layout is depicted in Figure 1. Participants were instructed to keep their eyes on the fixation cross and to minimise blinks during the trial.

S1 were coloured thin arrows. There were three preparation conditions: simple (SIM), complex (COM) and partial information (PIN). In the SIM and COM conditions full information about the upcoming movement was provided with arrow direction and colour 100% predictive. Arrow direction indicated whether to use the left or the right hand and colour indicated whether the finger-thump opposition sequence would be simple or complex. Simple movements were six repetitions of an index finger to thumb opposition. Complex movements were a sequence of thumb-finger oppositions: index finger twice, middle finger once, ring finger twice, little finger once. In the PIN condition arrow direction was predictive (i.e. left or right hand) but colour was uninformative. A simple or complex movement followed with equal likelihood in the PIN condition. The assignment of colour to preparation condition was counterbalanced across participants. An additional control condition (REST) was used where S1 and S2 were white arrows, which pointed inward toward each other. Participants were instructed simply to watch the screen and remain motionless during these trials. All stimuli were presented centrally for a duration of 150 ms, the fixation cross remained on-screen throughout stimulus presentation.

S2 were coloured block arrows. The direction of the arrows again specified movement with the left or right hand. Colour indicated to perform or imagine either a simple or complex movement. Movement duration was two seconds, after which a red square indicated that movement should be stopped. Participants were instructed to strictly adhere to the stop signal.

In a five minutes training period, participants familiarised themselves with the stimulus-response combinations and practised the timing of executing/imaging movements. Prior to the imagination sessions, participants were further trained to perform the imagination task without inducing muscle contractions. Briefly, the training procedure was firstly for participants to execute the task in response to the various stimuli to get used to the paradigm and then secondly to practise imagining the movements in response to the same stimuli. During this point participants could see their electromyogram (EMG) trace at high sensitivity on the screen and were asked to practice until they could imagine the task without an EMG response. They were reminded to use kinaesthetic imagery rather than visual imagery during this training period and in the experiment. The training length was a minimum of 5 minutes, but some participants wanted longer to feel that they were comfortable with the imagination task.

All participants participated in both the imagination and the execution session. The order of the sessions was counterbalanced across participants. Both experimental sessions comprised eight blocks of trials with 16 trials of each preparation condition (split equally into left and right-hand movements) and eight rest trials presented in a random order. To control for attention an additional four catch trials were presented at random times within each block. Here, a question mark was presented instead of the red square following the imagination period. This instructed participants to press a key with the finger they last imagined to be in contact with the thumb. For consistency, catch trials were also included in the execution session. As the study aimed on preparatory brain activity only and behavioural data were not considered of relevance for the interpretation of the results, no additional behavioural data were collected (for recent studies adopting a similar approach see [14], [15]). EMG was recorded throughout the experiment to ensure that subjects adhered to task instructions and did not move their hand in the preparation phase and during imagery of the movement.

### Electrophysiological Recording and Processing

EEG signals were continuously recorded from Ag/AgCl electrodes using a 64-channel QuickAmp amplifier (Brain Products; http://www.brainproducts.com). Electrodes were positioned according to the international 10–10 system. Electrodes were recorded against an average reference calculated by the amplifier hardware. Vertical (VEOG) and horizontal (HEOG) electrooculographic signals were recorded bipolarly. EMG was recorded bipolarly from electrodes positioned over the right and left forearm (flexor digitorum). Data were sampled at 500 Hz and recorded in DC mode. Electrode impedances were kept below 5 kOhm. Data were analysed offline using BrainVision Analyzer (Brain Products; www.brainproducts.com) software. EMG was digitally filtered (high-pass 30 Hz, low-pass 50 Hz, 12 dB/oct). Data were segmented into 8 s epochs from 2500 ms pre- to 5500 ms post-S2. Epochs were visually inspected and rejected if contaminated by artefacts. Additionally, epochs were rejected if EMG activity was present during the foreperiod or the imagination period. An automatic detection algorithm was used to determine the presence of EMG activity using a threshold method [17]. Per epoch, the mean and standard deviation (SD) of EMG activity in the baseline period (1 s prior to S1) were calculated. A sliding 25 ms window was used in the test period to calculate mean EMG activity. If mean activity within the window lay outside a specified multiple of SDs from the baseline mean, this was considered significant EMG activity. The SD multiple in the calculation (range 2–3) was tailored for each participant by calibrating the algorithm using their overt execution period. EMG activity flagged by this algorithm was also manually checked for false positives. On completion of artefact rejection, a minimum of 77% of trials were retained for further analyses, yielding, on average, 98 epochs per preparation condition (split equally into left and right-hand trials) and 49 rest epochs per participant. Eye-related artefacts were removed from EEG signals using independent component analysis [18].

For further analysis, epochs were digitally filtered (high-pass 0.01 Hz, low-pass 25 Hz, 24dB/oct), averaged and baseline-corrected using a 200 ms period pre-S1 to yield stimulus-locked ERPs for each condition. Grand means were calculated for visual inspection and the selection of relevant time windows for further statistical analysis.

### Data Analysis

CNV was statistically analysed using mean amplitudes pooled from fifteen pre-selected electrode sites over sensorimotor areas (FC1 to 4, FCz, C1 to 4, Cz, CP1 to 4, CPz). Two 300 ms time windows were selected for analysis: early (700–1000 ms post-S1) and late (1000–1300 ms post-S1). Amplitude differences were tested using a three-way ANOVA with factors session (execution, imagination), time (early, late) and condition (REST, PIN, SIM, COM).

To test whether any differences were restricted to sensorimotor areas or extended to and might thus be influenced by parieto-occiptial regions, the same ANOVA was run for 17 parieto-occipital channels (P1 to P8, Pz, PO3, PO4, PO7, PO8, POz, O1, O2, Oz). Topographical analysis was performed by defining a 5×5 grid of electrodes over the centre of the head covering the peak distribution of CNV activity. This grid specified the electrode locations along the medial-lateral (X-) and the anterior-posterior (Y-) axis. The CNV time window (700 to 1300 ms post-S1) was sectioned into six consecutive 100 ms time windows. Several repeated measures ANOVAs were run. The first ANOVA was a five-way ANOVA aimed at testing topographical differences across conditions. Factors were condition (PIN, SIM, COM) by window (1–6) by session (execution/imagination) by X-axis (left lateral, left medial, midline, right medial, right lateral) by Y-axis (frontal, fronto-central, central, centro-parietal, parietal). Topographical differences across sessions within each condition were assessed with three further four-way ANOVAs comprising the factors window (1–6) by session (execution/imagination) by X-axis (left lateral, left medial, midline, right medial, right lateral) by Y-axis (frontal, fronto-central, central, centro-parietal, parietal). Normalised data using the vector length method [19] were used to account for amplitude differences in the two sessions.

To test whether the observed pattern of results was due to a different degree of small anticipatory responses that were not detected by the EMG rejection algorithm, significant CNV effects were followed up by EMG analyses. For statistical analysis, absolute values of EMG data were calculated for each participant and performing hand, and averaged for the late time window (1000–1300 ms), for which CNV differences were observed. For the imagination-execution comparison, data were averaged across all imagination and execution conditions (SIM, COM, and PIN respectively) separately for each hand and subjected to two-tailed t-tests.

To explore whether the observed pattern of results might have been due to the participants not being able to make a distinction between imagery of the simple and the complex task and effectively perform the same in either condition during the imagination period we tested in an exploratory analysis the event-related desynchronisation (ERD) in the beta frequency range following S2. This beta suppression is thought to reflect the recruitment of populations of neurons in the motor cortex and therefore to index cortical activity during a task [20]. ERD/ERS power changes were calculated according to the classical method [20]. Epochs were digitally filtered in the 17–26 Hz range (48DB/oct). Samples were then squared and averaged across trials. Power amplitudes for each electrode (E) were normalised by calculating percentage power changes as a ratio of the average power in 1500 ms baseline period (B) in the REST condition (3000–4500 ms post-S2). Thus %ERD/ERS was calculated as (E-B)/Bx100. Negative percentages indicate a power decrease (desynchronisation) and positive percentages indicate a power increase (synchronisation). ERD/ERS power differences were analysed using a 1 s time window (500–1500 ms post S2). Selection of this time window minimised the contribution of S2 presentation to the analysed power values. Data pooled from 15 electrode sites over sensorimotor brain areas (FC1-4, FCz, C1-4, Cz, CP1-4, CPz) were statistically compared in a 2-way repeated measures ANOVA with factors condition (REST, SIM, COM) and session (execution, imagination).

In all analyses ANOVAs were Huynh-Feldt adjusted where necessary. Reported are corrected degrees of freedom and corrected F- and p-values.

## Results

### CNV

**Figure 2:**
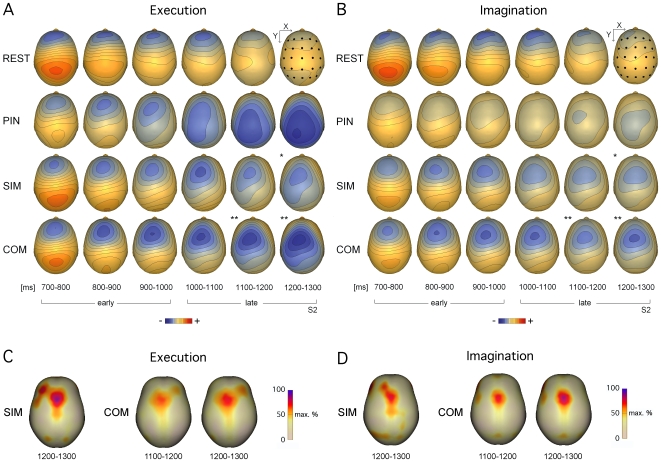
(A, B) Grand average topographical maps and (C, D) minimum norm solutions for the maps in A and B for which significant differences between execution and imagination sessions were found. Results are shown for (A) execution and (B) imagination sessions. Maps are shown for respectively six consecutive 100 ms time windows during the early and late CNV projected onto a realistic head surface for each condition. Scale is .5µV/step. Plotted electrodes define a 5 x 5 grid for formal testing of topographical differences. Starred maps indicate significant differences between execution and imagination sessions at the .05 (*) and .01 (**) level (uncorrected). Solutions are shown for execution (C) and imagination (D) sessions.

In both sessions, the foreperiod was characterised by a slow-rising negativity peaking at S2 presentation. This negativity was observed over central electrodes in all conditions (Figure 2). At S2 presentation the REST condition amplitude stabilised at baseline whilst SIM, PIN and COM conditions showed greater negative amplitudes respectively. Amplitudes in these conditions were attenuated in the imagination session compared with the execution session, particularly in the late time window.

**Figure 3:**
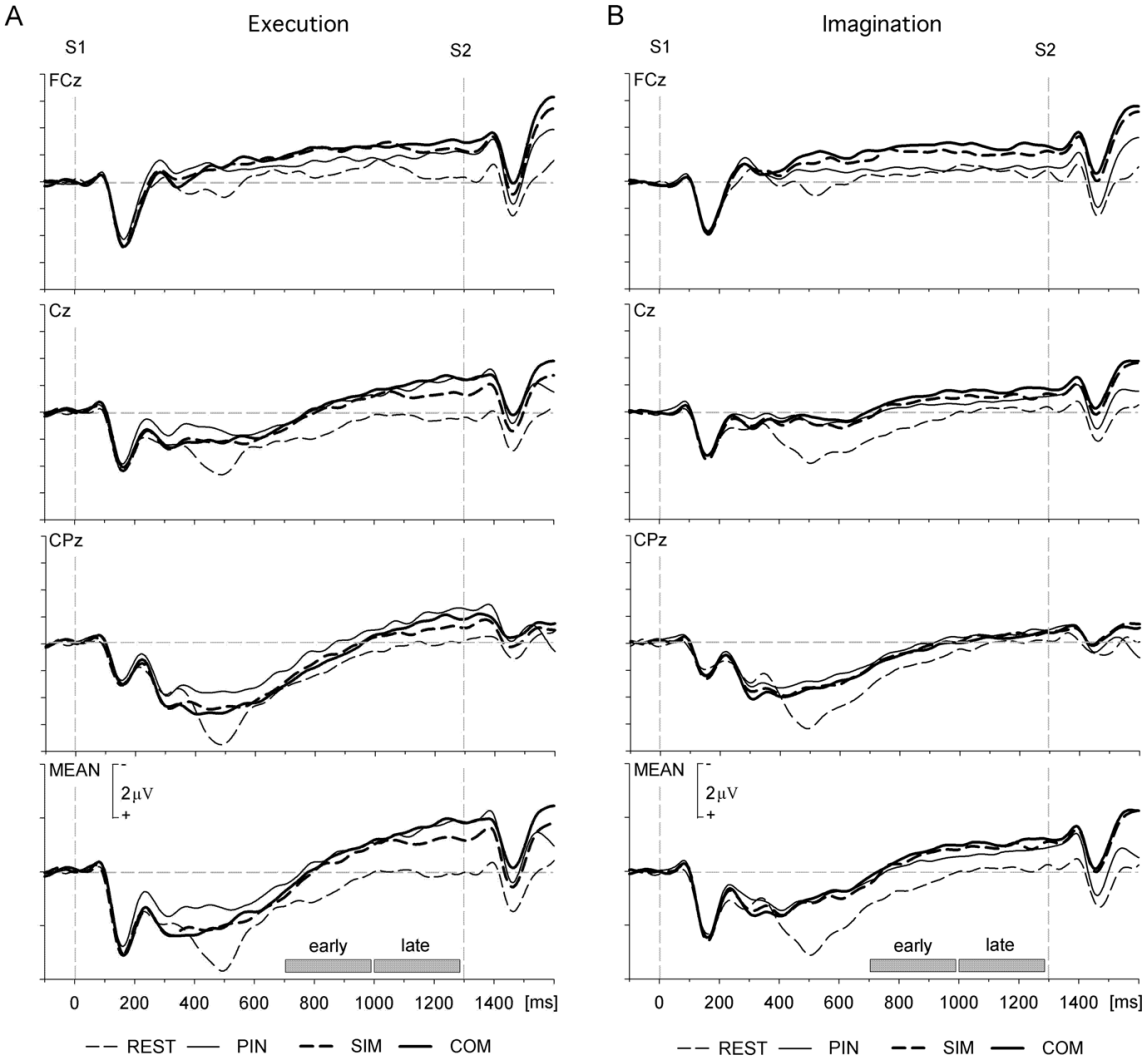
Grand average CNV amplitudes. Results are shown for (A) execution and (B) imagination sessions. (A, B) Amplitudes are pooled over fifteen central electrode sites (FC1 to 4, FCz, C1 to 4, Cz, CP1 to 4, CPz) in each condition for early and late time windows. Significant differences are indicated at the .05 (*), .01 (**) and .005 (***) level. Error bars show ±1 standard error.

The ANOVA for the sensorimotor area revealed a main effect of time [F(1, 11)  = 34.9, p<.001] with greater amplitudes in the late CNV window as compared to the early window [−0.11 µV (early) vs. −0.87 µV (late)]. A session x time interaction [F(1, 11)  = 24.1, p<.001] indicated that, across conditions, the change in amplitude over time was significantly different in the two sessions, with an attenuation of the late CNV amplitude in the imagination session [−0.70 µV (late imagination) vs. −1.05 µV (late execution)]. There was also a three-way interaction of session x time x condition [F(3, 33)  = 7.5, p<.01] which revealed that the pattern of condition differences changed across time in a significantly different way in the execution and imagination sessions. In the execution session, the REST condition differed significantly from all other conditions in both time windows [Fs(1, 11) >15.0, p<.005] while SIM and COM were significantly different in the late CNV only [F(1, 11)  = 9.1, p<.05]. In the imagination session, all conditions differed significantly from the REST condition in both time windows [Fs(1, 11) >5.0, p<.05], while PIN, SIM and COM showed no significant amplitude differences from each other. This result is illustrated in Figure 3. Table 1 gives the corresponding exact CNV values for both sessions and all four conditions. The ANOVA for the parieto-occipital region similarly revealed a significant session x time interaction [F(1, 11)  = 6.5, p<.05]. Post-hoc comparisons did however not show the significant difference of amplitudes in the late time window observed for the sensorimotor CNV [0.21 µV (late imagination) vs. −0.1 µV (late execution)], nor a significant difference for the early time window [0.44 µV (early imagination) vs. 0.39 µV (early execution)].

**Table 1:** CNV amplitudes for both sessions and all conditions for the early (700 to1000 ms) and late (1000 to 1300 ms) time windows.

		Early window	Late window
execution	REST	0.74 µV	0.05 µV
	PIN	−0.47 µV	−1.52 µV
	SIM	−0.20 µV	−1.15 µV
	COM	−0.37 µV	−1.60 µV
imagination	REST	0.61 µV	−0.09 µV
	PIN	−0.23 µV	−0.70 µV
	SIM	−0.42 µV	−0.92 µV
	COM	−0.55 µV	−1.07 µV

**Figure 4:**
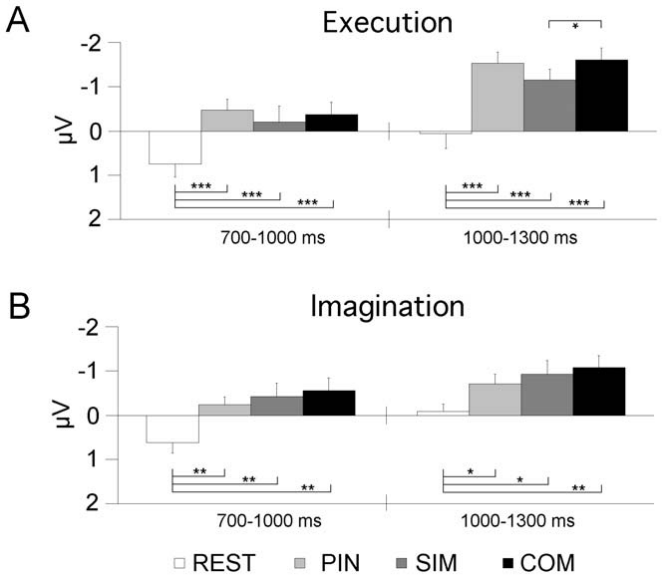
Grand average foreperiod CNV. Results are shown for (A) execution and (B) imagination sessions. (A, B) Shown is the CNV at electrode sites FCz, Cz and CPz and pooled over fifteen central electrode sites (Mean; FC1 to 4, FCz, C1 to 4, Cz, CP1 to 4, CPz) in each condition. Shaded bars indicate the early and late time windows selected for formal analysis of CNV.

Topographical analysis of the CNV showed an early frontocentral bilateral distribution that, in the execution session, shifted posterior towards a more centroparietal distribution at the end of the foreperiod. In the imagination session this posterior shift was less pronounced (Figure 4 A, B). In both sessions, the CNV was more centroparietally distributed in the PIN as compared to the SIM and COM conditions.

The 5-way ANOVA indicated a significant condition by Y interaction [F(3.8, 42)  = 4.4, p<.01]. This interaction was neither affected by the factor window (interaction window x condition x Y n.s.) nor the factor session (interaction session x condition x Y n.s.). Post-hoc analyses confirmed that the CNV in the PIN condition was significantly more centroparietally distributed than the CNVs in SIM and COM conditions (significant main effects of condition for centroparital and parietal regions, all F≥5.3, all p<.05).

In all three conditions (PIN, SIM, COM) there was a significant window by Y interaction confirming that, across sessions, the distribution shifted along the anterior/posterior axis towards the end of the foreperiod [all F≥11.9, all p<.001]. In addition, in the SIM and COM conditions, there was a significant window by session by Y interaction indicating that the change in distribution over time was different for execution and imagination in these conditions [all F≥2.8, all p<.05]. Post-hoc analyses of the window by session by Y interactions revealed that there were three time windows in which the topographical distribution differed along the Y axis between the execution and imagination sessions: In the SIM condition the 1.2–1.3 s window [F(1.5, 16)  = 5.1, p<.05], and in the COM condition both the 1.1–1.2 and 1.2–1.3 s windows [all F≥7.7, all p<.005].

### EMG

**Figure 5:**
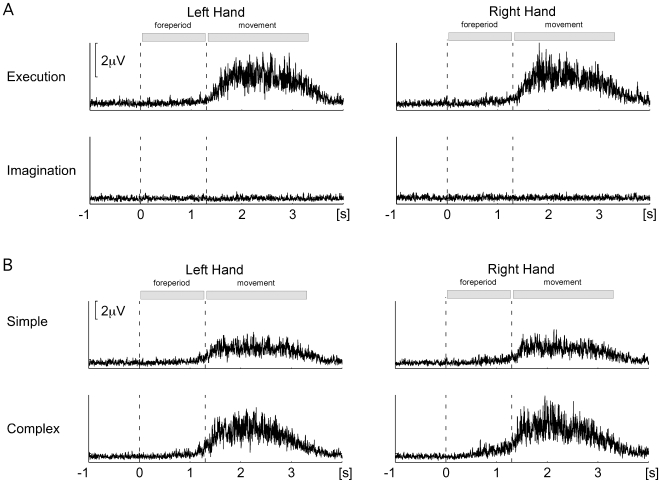
Grand average EMG data. (A) EMG data for the imagination (IM) and execution (EX) sessions averaged across partial information (PIN), simple (SIM), and complex (COM) conditions and separately for left and right hand trials. (B) EMG data for the SIM and COM conditions of the EX session separately for left and right hand trials.

Figure 5 shows grand mean EMG raw data for the imagination and execution sessions (Figure 5A) and the SIM and COM conditions in the execution session (Figure 5B). No significant differences between imagination and execution sessions were observed (both p>0.39). The SIM-COM comparison was restricted to the execution session. Again, neither for the left nor for the right hand did we observe significant differences (both p>0.35).

### ERD

The results of this exploratory analysis showed a significant main effect of condition [F(2,22)  = 15.3, p<0.0001] but no interaction effect (p = 0.16), indicating that the observed pattern of condition differences was present in both sessions. The main effect of session was not significant (p>0.068). Contrast analyses revealed significant higher ERD for both movement conditions as compared to the REST condition (execution: Fs(1,11) >11, p<0.01, imagination Fs(1,11)>8, p<0.05]. Also, ERD was significantly lower in the SIM as compared to the COM conditions for both execution [F(1, 11)  = 5.4, p<.05] and imagination [F(1, 11)  = 6.8, p<.05].

## Discussion

The present study compared the effects associated with advance information on simple and complex sequential finger movements during the preparation of covert and overt motor action. Importantly, both tasks elicited a late CNV suggesting the engagement of preparatory processes in both tasks. The late CNV amplitude was thereby attenuated in the imagery task as hypothesised. The data further confirmed our expectation that complex movements were associated with greater CNV amplitudes in the execution task. In contrast to our prediction, however, no reduction in CNV amplitude was found for partial advance information. In the imagery task, neither task complexity nor the degree of advance information modulated the late CNV amplitude.

The most important novel aspect of this study is the direct comparison of the preparation for imagined and executed movements under varying degrees of task complexity and advance information. Such a direct comparison utilizing the CNV as an indicator of motor preparation has to our knowledge not been published before. Critically, the execution and imagery conditions displayed greater CNV amplitudes than the REST condition which clearly demonstrates preparation-related activity during movement imagination that is over and above the activity associated with stimulus anticipation and general task arousal. To this end our results confirm previous reports [11]–[13] but also provide more direct evidence for preparation-related activity in movement imagination.

The topographical analysis of foreperiod activity revealed differences between execution and imagery sessions for the last part of the foreperiod interval, with a more posterior distribution prior to movement execution. This, and the general attenuation of the CNV amplitude in the imagery session, is consistent with the idea of a lesser contribution of more posterior (primary) motor areas to the preparatory activity in the imagery session e.g. [15]. It also mirrors the results obtained by Caldara et al. [13] who found that a topographical map representing primary motor activity fitted execution preparatory activity significantly better than equivalent imagery activity. It is not consistent though with the results of an analysis of lateralised motor and non-motor preparatory activity of the same data set where no attenuation of the LRP for movement imagination was found [14]. The source of the LRP is commonly attributed to primary motor cortex and lateral pre-motor areas [21]–[25]. Thus, results rather suggest an underlying attenuation of activity in other regions of the widespread neural network assumed to contribute to the CNV that includes, beside primary motor and pre-motor areas, supplementary motor, primary sensory, and prefrontal cortical areas, but also temporal and occipital regions [7], [26]. A significant role of sensory areas for the evolution of the observed effects seems unlikely. Which of the more cognitive parts of the network are of particular relevance for the topographical and amplitude effects cannot be answered at this stage, but intra-cranial recordings or the selective functional silencing of particular brain regions using transcranial magnetic stimulation (TMS) or transcranial direct current stimulation (tDCS) will help to answer this question.

In contrast to our expectations, variations in advance movement information only modulated preparatory activity in the overt movement condition, not the covert, imagery, condition. The simplest explanation for this lack of late CNV differences is that participants did not make enough of a distinction between imagery of the simple and the complex task as they did not have any benefit from it and effectively performed the same task in either condition during the imagination period. Such a strategy would result in a reduced effect size in the late CNV and a power problem fro detecting CNV differences, strengthened by the overall attenuation of the CNV in the imagery condition. Even though it is impossible to completely rule out this explanation when both preparation and movement are covered, several arguments make a case against a general lack of distinction between the simple and the complex task in the imagination session. The exploratory analysis of the ERD in the beta frequency range following S2 indicated a significantly lower ERD in the SIM as compared to the COM conditions also for the imagination session, which clearly indicates that participants did make a distinction between the simple and the complex tasks, at least during the post-S2 interval. Importantly, as in the imagination session both pre-S2 and post-S2 intervals were covered stages it also suggests that participants did make this distinction in the pre-S2 interval as well. This line of argumentation is also supported by the results of an fMRI study in which different patterns of brain activity were observed during the imagination of simple and complex movements [27]. Secondly, the interspersed catch trials ensured that participants performed either the simple or the complex task also in the imagery condition.

Another account of the lack of CNV differences could be that the general attenuation of the CNV in the imagery session reduces the effect size of any condition difference, rendering it non-significant. However, if this were the case one would still expect the same pattern of results across sessions. This, however, is not the case with CNV amplitude in the execution session being largest for the COM condition, followed by PIN and then SIM conditions as compared to the imagination session where the largest CNV amplitude was observed for COM, followed by SIM and then PIN conditions (cf. Figure 3). The different pattern of results could be interpreted as indicating that in addition to a moderate imagination-related general attenuation, there is some attenuation that is specific to the condition in which a task-specific preparation is not possible (PIN). This attenuation is likely not to be due to an attenuation of lateralised motor and attention-directing responses though, which have been found to be comparable for imagination and execution if pre-cues allow only the selection of response hand but not the preparation of a particular task [14], [15]. Hence, if the PIN-specific attenuation is not a spurious effect, an explanation that remains is that it is due to differences in cognitive strategies in the preparation of incompletely specified imagined and executed movements. It is for instance conceivable that in the imagination session subjects employed less anticipatory attention to the S2 stimulus.

As a final remark, a surprising result of the present study was that in the execution task the PIN condition showed a CNV amplitude comparable to the COM condition. Previous studies have found that the late CNV amplitude increases with the amount of information provided by S1 [9], [10], [28]. On the basis of this evidence, one would predict that the PIN condition (unspecified complexity) would show a lesser CNV than the SIM condition that fully specifies the upcoming movement. A potential though speculative explanation for the PIN condition result is that participants adopted a ‘worst-case scenario’ preparatory strategy whereby, in the absence of complexity information, participants default to preparing the most difficult response. If this were the case one would expect identical results for the COM and PIN conditions. Topographical data do not confirm this expectation however, with quite different foreperiod topographies in the COM (and SIM) and PIN conditions. The difference in topography suggests a qualitative difference between COM/SIM and PIN conditions, whose nature might be that in the PIN condition S2 is highly relevant, as it additionally signals what has to be done. This might result in a modulation of CNV activity related to anticipatory attention that overshadows the motor preparation aspect. It has been previously noted that the CNV reflects both motor preparation and anticipatory attention who overlap in time and in their electrophysiological reflection [29]. Interestingly, even though the PIN condition did not behave as expected in terms of CNV amplitude, CNV topographies in this condition were very comparable in the imagination and execution sessions. This supports the notion of similar neural mechanisms underlying overt and covert movements [1].

In sum, the present study directly compared the preparation for overt and covert movements within the same experiment manipulating information content and task complexity. Thereby the study aimed to affirm the notion of functional similarity of the two modes of movement. Results clearly show that the preparation for imagined movements is associated with systematic preparation-related activity that is over and above the activity associated with stimulus anticipation and general task arousal. Results also demonstrate that motor imagery and execution share common features and that these similarities extend into the mechanisms subserving the processes of motor preparation. However, our findings also demonstrate that preparation for these two modes of movement cannot be exactly equated. Differences in preparation for simple and complex movements in the late part of the preparation period observed in the execution session were absent for motor imagery. This is probably due to an attenuation of activity in the cognitive aspects of the widespread network generating the CNV. Another likely contributor are different cognitive strategies in the preparation of executed and imagined movements. Future research should now focus on elucidating which aspects and generators of the CNV are at the root of the CNV differences observed here.
